# The path from early- to late-liver stage arresting genetically attenuated parasites as a malaria vaccination strategy

**DOI:** 10.1038/s41541-025-01265-z

**Published:** 2025-10-08

**Authors:** O.A.C. Lamers, B.M.D. Franke-Fayard, M. Roestenberg, J.M.M. Krol

**Affiliations:** Leiden University Center for Infectious Diseases, Leiden University Medical Center, Leiden, The Netherlands

**Keywords:** Infection, Malaria, Live attenuated vaccines

## Abstract

Genetically attenuated parasites (GAPs) that arrest during liver stage development have shown significant potential as malaria vaccines. Compared to *Plasmodium falciparum* GAPs that arrest after 24–48 h (early-arresters), parasites arresting after 6–7 days (late-arresters) have shown superior efficacy, highlighting the importance of liver stage immunity in promoting sterile protection. Here, we describe GAPs tested in humans and the pre-clinical research that led to their creation. We discuss safety and efficacy of existing GAPs with particular focus on their large-scale implementation as malaria vaccines.

## Main Text

Decades of malaria research have led to WHO recommendations for two malaria vaccines, RTS,S and R21, both based on the circumsporozoite protein (CSP), the most abundant protein on the surface of *Plasmodium* sporozoites^1–3^. The mechanism of action of these subunit vaccines is thought to be solely mediated by antibodies preventing sporozoite invasion of hepatocytes^4^. Hence, efficacy, defined as the percentage reduction of malaria incidence in vaccinated trial participants compared to unvaccinated controls^5,6^, of both RTS,S and R21, is variable (30–80%) and declines over time with waning antibody titres^1,7,8^. While approval of the two above-mentioned vaccines certainly represents an important step in the fight against malaria, it is insufficient to reach the WHO goal of reducing mortality by 90% in 2030^9^.

Whole sporozoite (WSp) vaccine candidates consist of live *Plasmodium falciparum* (*Pf*) parasites that have been attenuated by either radiation, co-administration of chemoprophylaxis or genetic modification^10^. Protection, defined as the parasite’s ability to prevent the onset of malaria after exposure to unattenuated parasites, can be tested by means of controlled human malaria infections (CHMIs). During CHMI, live unattenuated malaria parasites are administered in a controlled setting to healthy participants^11^.

Though radiation-attenuated sporozoites (RAS) achieve high protection when administered in high doses to malaria-naive individuals^12^, in endemic areas their performance remains suboptimal^13–15^. Chloroquine chemoprophylaxis with *Pf* sporozoites (CPS) is the most potent WSp vaccination strategy to date, inducing 100% protection in malaria-naive individuals after mosquito bite or intravenous immunization^16,17^. However, the requirement for drugs and the administration of unattenuated parasites to healthy individuals makes this method unattainable for large-scale implementation.

Genetically attenuated parasites (GAPs) are another type of WSp vaccine where attenuation is achieved by gene deletion. Genetic modification allows the generation of a homogeneous parasite population that arrests at a targeted life cycle stage to induce an immune response^18^.

In the following, we will review the current global portfolio of GAP vaccine candidates which arrest during liver stage development with a particular focus on those that have or will soon be tested in clinical trials (Table 1, Table 2 and Table 3). Depending on which genes have been removed from the *Plasmodium* genome, currently existing liver stage GAPs fall into two distinct phenotypic categories: arresting either early or late during liver stage development. GAPs will be described based on this categorization and in order of creation, starting from early- and ending with late-arresting GAPs.

**Table 1:** Overview of studies on murine liver-arresting GAPs

Author	Parasite	Mother line
*Murine liver-arresting GAPs that did not progress to clinical studies*		
Mueller et al., 2005^19^	*PbΔuis3*	*Pb* NK65
Mueller et al., 2005^20^	*PbΔuis4*	*Pb* NK65
Tarun et al., 2007^23^	*PyΔuis3*	*Py* 17XNL
	*PyΔuis4*	*Py* 17XNL
Jobe et al., 2007^24^	*PbΔuis3Δuis4*	*Pb* NK65
Yu et al., 2008^29^	*PbΔFabl*	*Pb* ANKA
Butler et al., 2011^28^	*PyΔFabB/F*	*Py* 17XNL
Annoura et al., 2012^34^	*PbΔFabB/F*	*Pb* ANKA
*Murine liver-arresting GAPs that progressed to clinical studies*		
Van Dijk et al., 2005^33^	*PbΔp52*	*Pb* ANKA
Labaied et al., 2007^31^	*PyΔp52Δp36*	*Py* 17XNL
Aly et al., 2011^39^	*PyΔsap1*	*Py* 17XNL
Kublin et al., 2017^41^	*PyΔp52Δp36Δsap1*	*Py* 17XNL
Annoura et al., 2012^34^	*PbΔp52Δp36*	*Pb ANKA*
Annoura et al., 2014^43^	*PbΔb9*	*Pb* ANKA
Van Schaijk et al., 2014^44^	*PbΔb9Δslarp*	*Pb* ANKA
Dankwa et al., 2016^47^	*PyΔmei2*	*Py* 17XNL
Franke-Fayard et al., 2022^48^	*PbΔmei2*	*Pb* ANKA
Goswami et al., 2024^54^	*PyΔmei2Δlinup*	*Py* 17XNL

**Table 2:** Overview of studies in humans using liver-arresting GAPs

Author	Parasite	Mother line	Number of participants	Location
Spring et al., 2013^35^	*PfΔp52Δp36* (2KO)	*Pf*NF54	6 participants	Silver Spring, USA
Kublin et al., 2017^41^	*PfΔp52Δp36Δsap1* (3KO)	*Pf*NF54	10 participants	Seattle, USA
Murphy et al., 2022^42^	*PfΔp52Δp36Δsap1* (3KO)	*Pf*NF54	Cohort 1: 10 participants Cohort 2: 6 participants Control: 12 participants	Seattle, USA
Roestenberg et al., 2020^45^	*PfΔb9Δslarp* (GA1)	*Pf*NF54	Phase 1: 19 participants Phase 2: 26 participants Control: 9 participants	Leiden and Nijmegen, The Netherlands
Lamers et al., 2024^51^	*PfΔb9Δslarp* (GA1) *PfΔmei2* (GA2)	*Pf*NF54	Phase 1: 20 participants Phase 2: 20 participants Control: 3 participants	Leiden and Nijmegen, The Netherlands
Roozen et al., 2025^52^	*PfΔmei2* (GA2)	*Pf*NF54	Cohort 10 participants Control: 5 participants	Leiden, The Netherlands

**Table 3:** Overview of test status of all GAPs intended for clinical testing

Parasite	Approved for clinical testing	Test-status	Further development
*PfΔp52Δp36* (2KO)	Yes	Tested	Not planned
*PfΔp52Δp36Δsap1* (3KO)	Yes	Tested	Not planned
*PfΔb9Δslarp* (GA1)	Yes	Tested	Not planned
*PfΔmei2* (GA2)	Yes	Tested	Planned
*PfΔmei2Δlinup* (LARC2)	Yes	In progress	Not applicable yet

## GAPs that did not progress to clinical testing

### Double knock-out based on Δuis3Δuis4

The search for essential genes for *Plasmodium* liver stage development identified upregulated in infective sporozoites (*uis*) genes as initial targets (Table 1)^19,20^. These genes are upregulated in salivary gland sporozoites, yet are translationally repressed so that protein expression occurs upon infection of hepatocytes^21,22^. Single deletion *P. berghei* (*Pb*) mutants *PbΔuis3* and *PbΔuis4* showed attenuation during liver stage development^19,20^.

No breakthrough blood stage infections were observed for *PbΔuis3* parasites when inoculated into rats in doses of up to 1 × 10⁵ sporozoites^19^. In C57BL/6 mice, immunization with 1 × 10⁴ *PbΔuis3* plus two boosts of 2.5 × 10⁴ provided complete protection against challenge with 1 × 10⁴ wild-type (WT) parasites (Table 4). A reduced dose (twice 1 × 10⁴) still protected 70% of mice^19^. The *P. yoelii* (*Py*) analog also conferred 100% protection after three immunizations with 1 × 10^4^ sporozoites (Table 4)^23^.

**Table 4:** Overview of the protection rate of liver-arresting GAPs in mice

Author	Parasite	Mice	Dose	Challenge	Protection
Mueller et al., 2005^19^	*PbΔuis3*	C57BL/6	5 × 10^4^ and then 2.5 × 10^4^ IV 14 and 21 days after first immunization	1 × 10^4^ IV 7 days later	10/10 (100%)
	*PbΔuis3*	C57BL/6	1 × 10^4^ and then 1 × 10^4^ IV 14 and 21 days after first immunization	1 × 10^4^ IV 7 days later	10/10 (100%)
	*PbΔuis3*	C57BL/6	5 × 10^4^ and then 2.5 × 10^4^ IV 34 and 45 days after first immunization	1 × 10^4^ IV 30 days later	5/5 (100%)
	*PbΔuis3*	C57BL/6	1 × 10^4^ and then 1 × 10^4^ IV 34 and 45 days after first immunization	1 × 10^4^ IV 30 days later	5/5 (100%)
	*PbΔuis3*	C57BL/6	5 × 10^4^ and then 5 × 10^4^ 14 days and 1 × 10^4^ IV 21 days after first immunization	10 mosquitos/mouse/10 min. 38 days later	5/5 (100%)
	*PbΔuis3*	C57BL/6	1 × 10^4^ and then 1 × 10^4^ IV 14 days and 1 × 10^4^ 21 days after first immunization	10 mosquitos/mouse/10 min. 38 days later	5/5 (100%)
	*PbΔuis3*	C57BL/6	1 × 10^4^ IV twice at 14-day intervals	1 × 10^4^ IV 7 days later	7/10 (70%)
	*PbΔuis3*	C57BL/6	5 × 10^4^ and then 2.5 × 104 IV 14 and 21 days after immunization	1 × 10^4^ blood stage IV 30 days later	0/5 (0%)
	*PbΔuis3*	C57BL/6	1 × 10^4^ and then 1 × 104 IV 14 and 21 days after immunization	1 × 10^4^ blood stage IV 30 days later	0/5 (0%)
Mueller et al., 2005^20^	*PbΔuis4REP*	C57BL/6	5 × 10^4^ and then 2,5 × 104 IV twice at 14-day intervals	5 × 10^4^ IV 10 days later	8/8 (100%)
	*PbΔuis4REP*	C57BL/6	1 × 10^4^ IV three times at 14-day intervals	5 × 10^4^ IV 10 days later	8/8 (100%)
	*PbΔuis4REP*	C57BL/6	1 × 10^4^ IV three times at 14-day intervals	1 × 10^4^ IV blood stages 34 days later	0/4 (0%)
Jobe et al., 2007^24^	*PbΔuis3Δuis4*	C57BL/6	7.5 × 10^4^ and then 2 × 10^4^ IV at 7-day intervals	1 × 10^4^ IV 7 days later	27/27 (100%)
			NA	1 × 10^4^ IV rechallenge 180 days later	6/6 (100%)
	*PbΔuis3Δuis4*	C57BL/6	1 × 10^4^ IV three times at 14-day intervals	1 × 10^4^ IV 7 days later	14/14 (100%)
	*PbΔuis3Δuis4*	C57BL/6	1 × 10^4^ IV three times at 14-day intervals	1 × 10^4^ IV 118 days later	14/14 (100%)
Tarun et al., 2007^23^	*PyΔuis3*	BALB/c	5 × 10^4^ IV once	1 × 10^4^ IV 7 days later	0/4 (0%)
	*PyΔuis3*	BALB/c	5 × 10^4^ IV twice at 14-day intervals	1 × 10^4^ IV 7 days later	4/4 (100%)
	*PyΔuis3*	BALB/c	1 × 10^4^ IV twice at 14-day intervals	1 × 10^4^ IV 7 days later	4/4 (100%)
	*PyΔuis3*	BALB/c	1 × 10^4^ IV twice at 14-day intervals	1 × 10^4^ IV 30 days later	4/4 (100%)
	*PyΔuis3*	BALB/c	1 × 10^4^ IV three times at 14-day intervals	1 × 10^4^ IV 7 days later	4/4 (100%)
	*PyΔuis3*	BALB/c	1 × 10^4^ IV three times at 14-day intervals	1 × 10^4^ IV 60 days later	4/4 (100%)
	*PyΔuis3*	BALB/c	1 × 10^4^ IV three times at 14-day intervals	1 × 10^4^ IV 180 days later	8/12 (67%)
	*PyΔuis4*	BALB/c	5 × 10^4^ IV once	1 × 10^4^ IV 7 days later	4/4 (100%)
	*PyΔuis4*	BALB/c	5 × 10^4^ IV once	1 × 10^4^ IV 30 days later	4/4 (100%)
	*PyΔuis4*	BALB/c	5 × 10^4^ IV twice at 14-day intervals	1 × 10^4^ IV 7 days later	4/4 (100%)
	*PyΔuis4*	BALB/c	1 × 10^4^ IV twice at 14-day intervals	1 × 10^4^ IV 7 days later	4/4 (100%)
	*PyΔuis4*	BALB/c	1 × 10^4^ IV three times at 14-day intervals	1 × 10^4^ IV 7 days later	4/4 (100%)
	*PyΔuis4*	BALB/c	1 × 10^4^ IV three times at 14-day intervals	1 × 10^4^ IV 60 days later	4/4 (100%)
	*PyΔuis4*	BALB/c	1 × 10^4^ IV three times at 14-day intervals	1 × 10^4^ IV 180 days later	8/8 (100%)
Butler et al., 2011^28^	*PyΔsap1*	Swiss Webster	2 × 10^4^ IV once	1 × 10^3^ IV > 80 days later	8/20 (40%)
			Boost with 2 × 10^4^ IV	1 × 10^3^ IV > 60 days later	8/19 (42%)
	*PyΔsap1*	C57BL/6	2 × 10^4^ IV once	1 × 10^3^ IV > 60 days later	Not determined
			Boost with 2 × 10^4^ IV 111 days later	1 × 10^3^ IV > 60 days later	0/10 (0%)
	*PyΔsap1*	BALB/c	1 × 10^4^ IV twice at 14-day intervals	1 × 10^4^ IV 30 days later	2/10 (20%)
			1 × 10^4^ IV three times at 14-day intervals	1 × 10^4^ IV 30 days later	10/10 (100%)
	*PyΔFabB/F*	Swiss Webster	2 × 10^4^ IV once	1 × 10^3^ IV > 80 days later	8/20 (40%)
			Boost with 2 × 10^4^ IV 111 days later	1 × 10^3^ IV > 60 days later	18/20 (90%)
	*PyΔFabB/F*	C57BL/6	2 × 10^4^ IV once	1 × 10^3^ IV > 60 days later	1/10 (10%)
			Boost with 2 × 10^4^ IV	1 × 10^3^ IV > 60 days later	20/20 100%
	*PyΔFabB/F*	BALB/c	1 × 10^4^ IV twice at 14-day intervals	1 × 10^4^ IV 30 days later	10/10 (100%)
	*PyΔFabB/F*	BALB/c	1 × 10^4^ IV three times at 14-day intervals	1 × 10^4^ IV 30 days later	10/10 (100%)
	*PyΔFabB/F*	BALB/c	1 × 10^4^ IV three times at 14-day intervals	1 × 10^4^ IV 30 days later	8/8 (100%)
			NA	1 × 10^4^ IV rechallenge 120 days later	8/8 (100%)
			NA	1 × 10^4^ IV rechallenge 210 days later	8/8 (100%)
	*PyΔFabB/F*	BALB/c	1 × 10^4^ IV three times at 14-day intervals	1 × 104 IV 14 days later	8/8 (100%)
			NA	1 × 10^4^ IV rechallenge 210 days later	8/8 (100%)
	*PyΔFabB/F*	BALB/c	1 × 10^4^ IV three times at 14-day intervals	1 × 10^4^ IV 100 days later	5/5 (100%)
	*PyΔFabB/F*	BALB/c	1 × 10^4^ IV three times at 14-day intervals	1 × 10^4^ IV 300 days later	8/8 (100%)
	*PyΔFabB/F*	BALB/c	5 × 10^4^ IV three times at 14-day intervals	1 × 10^4^ IV 210 days later	8/8 (100%)
	*PyΔFabB/F*	BALB/c	5 × 10^4^ ID three times at 14-day intervals	1 × 10^4^ IV 30 days later	5/5 (100%)
			NA	1 × 10^4^ IV 210 days later	5/5 (100%)
	*PyΔFabB/F*	BALB/c	5 × 10^4^ SC three times at 14-day intervals	1 × 10^4^ IV 30 days later	5/5 (100%)
			NA	1×10^4^ IV 210 days later	5/5 (100%)
	*PyΔFabB/F*	BALB/c	1 × 10^4^ IV three times at 14-day intervals	1 × 10^4^ *Pb* IV 210 days later (heterologous)	5/5 (100%)
Van Dijk et al., 2005^33^	*PbΔp52*	BALB/c	1 × 10^5^ IV once	5 × 10^4^ IV 10 days later	18/20 (90%)
	*PbΔp52*	BALB/c	5 × 10^4^ IV once	2,5 × 10^4^ IV 10 days later	15/15 (100%)
	*PbΔp52*	BALB/c	5 × 10^4^ IV once	1 × 10^4^ IV 10 days later	5/5 (100%)
	*PbΔp52*	BALB/c	5 × 10^4^ IV once	1 × 10^4^ IV 30 days later	5/5 (100%)
	*PbΔp52*	BALB/c	5 × 10^4^ IV once	1 × 10^4^ IV 60 days later	5/5 (100%)
	*PbΔp52*	BALB/c	5 × 10^4^ IV once	1 × 10^4^ IV 120 days later	5/5 (100%)
	*PbΔp52*	BALB/c	5 × 10^4^ IV once	1 × 10^6^ infected RBCs IV 10 days later	0/4 (0%)
	*PbΔp52*	C57BL6	5 × 10^4^ IV once	1 × 10^4^ IV 10 days later	0/1 (0%)
	*PbΔp52*	C57BL6	5 × 10^4^ and then 2 × 10^4^ IV at 7-day intervals	1 × 10^4^ IV 10 days later	1/4 (25%)
	*PbΔp52*	C57BL6	5 × 10^4^ once and then 2 × 10^4^ IV twice at 7-day intervals	1 × 10^4^ IV 10 days later	4/4 (100%)
	*PbΔp52*	C57BL6	5 × 10^4^ once and then 2 × 10^4^ IV twice at 7-day intervals	1 × 10^4^ IV 30 days later	5/5 (100%)
Labaied et al., 2007^31^	*PyΔp52Δp36*	BALB/c	1 × 10^4^ IV three times at weekly intervals	1 × 10^4^ IV 7 days later	8/8 (100%)
	*PyΔp52Δp36*	BALB/c	1 × 10^4^ IV three times at weekly intervals	1 × 10^4^ IV 30 days later	7/7 (100%)
	*PyΔp52Δp36*	BALB/c	1 × 10^4^ IV three times at 7-day intervals	15 mosquitoes/ mouse/ 10 min 7 days later	5/5 (100%)
Aly et al., 2011^39^	*PyΔsap1*	Swiss Webster	2 × 10^4^ IV four times at 14-day intervals	1 × 10^4^ IV 60 days later	5/5 (100%)
	*PyΔsap1*	BALB/c	1 × 10^4^ IV three times at 14-day intervals	1 × 10^4^ IV 90 days later	8/8 (100%)
	*PyΔsap1*	BALB/c	1 × 10^4^ IV three times at 14-day intervals	1 × 10^4^ IV 180 days later	7/7 (100%)
	*PyΔsap1*	BALB/c	1 × 10^4^ IV three times at 14-day intervals	1 × 10^4^ IV 270 days later	6/6 (100%)
	*PyΔsap1*	BALB/c	5 × 10^4^ IV three times at 14-day intervals	1 × 10^4^ IV 60 days later	5/5 (100%)
	*PyΔsap1*	BALB/c	1 × 10^5^ SC three times at 14-day intervals	1 × 10^4^ IV 60 days later	5/5 (100%)
	*PyΔsap1*	BALB/c	1 × 10^5^ IV four times at 14-day intervals	20 mosquitoes/ mouse/ 10 min 360 days later	5/5 (100%)
Kublin et al., 2017^41^	*PyΔp52Δp36Δsap1*	BALB/cJ	1 × 10^4^ IV twice at 14-day intervals	1 × 104 IV 7 days later	5/5 (100%)
	*PyΔp52Δp36Δsap1*	BALB/cJ	1 × 10^4^ IV twice at 14-day intervals	1 × 10^4^ IV 30 days later	5/5 (100%)
	*PyΔp52Δp36Δsap1*	BALB/cJ	1 × 10^4^ IV twice at 14-day intervals	1 × 10^4^ IV 180 days later	5/5 (100%)
Van Schaijk et al., 2014^44^	*PbΔb9*	BALB/c	1 × 10^3^ IV once	1 × 10^4^ IV 10 days later	8/10 (80%)
	*PbΔb9*	BALB/c	5 × 10^3^ IV once	1 × 10^4^ IV 10 days later	18/20 (90%)
	*PbΔb9*	BALB/c	1 × 10^4^ IV once	1 × 10^4^ IV 10 days later	10/10 (100%)
	*PbΔb9*	C57BL/6	5 × 10^4^ and then 2 × 10^4^ IV at 7-day intervals	1 × 10^4^ IV 10 days later	4/4 (100%)
	*PbΔb9*	C57BL/6	5 × 10^4^ and then 2 × 10^4^ IV at 7-day intervals	1 × 10^4^ IV 90 days later	5/5 (100%)
	*PbΔb9*	C57BL/6	5 × 10^4^ and then 2 × 10^4^ IV at 7-day intervals	1 × 10^4^ IV 180 days later	9/9 (100%)
	*PbΔb9*	C57BL/6	5 × 10^4^ and then 2 × 10 IV at 7-day intervals	1 × 10^4^ IV 365 days later	5/11 (46%)
	*PbΔb9Δslarp*	BALB/c	1 × 10^3^ IV once	1 × 10^4^ IV 10 days later	20/20 (100%)
	*PbΔb9Δslarp*	BALB/c	5 × 10^3^ IV once	1 × 10^4^ IV 10 days later	10/10 (100%)
	*PbΔb9Δslarp*	BALB/c	1 × 10^4^ IV once	1 × 10^4^ IV 10 days later	20/20 (100%)
	*PbΔb9Δslarp*	C57BL/6	5 × 10^4^ and then 2 × 10^4^ IV at 7-day intervals	1 × 10^4^ IV 180 days later	6/6 (100%)
	*PbΔb9Δslarp*	C57BL/6	1 × 10^3^ IV three times at 7-day intervals	1 × 10^4^ IV 10 days later	6/10 (60%)
	*PbΔb9Δslarp*	C57BL/6	1 × 10^4^ IV three times at 7-day intervals	1 × 10^4^ IV 10 days later	10/10 (100%)
Dankwa et al., 2016^47^	*PyΔmei2*	BALB/cJ	1 × 10^4^ IV three times, 35 days and then 45 days after the first immunization	1 × 10^4^ IV 46 days later	9/9 (100%)
			NA	1 × 10^4^ IV rechallenge 56 days later	9/9 (100%)
	*PyΔmei2*	BALB/cJ	1 × 10^4^ IV three times, 31 and 45 days after the first immunization	1 × 10^4^ IV 39 days later	12/12 (100%)
Goswami et al., 2024^54^	*PyΔmei2Δlinup*	BALB/cJ	1 × 10^4^ IV, twice at 1-month intervals	1 × 10^4^ IV 30 days later	10/10 (100%)
		BALB/cJ	2 × 10^4^ IM, twice at 1-month intervals	1 × 10^4^ IV 30 days later	7/10 (70%)
		BALB/cJ	5 × 10^4^ IV, twice at 1-month intervals	1 × 10^4^ IV 180 days later	9/10 (90%)
		BALB/cJ	5 × 10^4^ IV, twice at 1-month intervals	1 × 10^4^ IV 360 days later	6/10 (60%)
		BALB/cJ	5 × 104 cryopreserved IV	1 × 10^4^ IV 30 days later	10/10 (100%)
		BALB/cJ	1 × 10^4^ IV, 3 times at 1-month intervals	1 × 10^4^ IV blood stage parasites 30 days later	10/10 (100%)
		BALB/cJ	5 × 10^4^ cryopreserved sporozoites IV, 2 times at 15-day intervals	1 × 10^4^ cryopreserved sporozoites IV 25 days later	12/12 (100%)
		Swiss Webster (outbred)	5 × 10^4^ IV, at 2-month intervals	1 × 10^4^ IV 60 days later	7/10 (70%)

IV: intravenous, SC: subcutaneous, ID: intradermal, IM: intramuscular.

For *PbΔuis4* parasites, breakthroughs were observed in 59% of C57BL/6 mice upon administration of 5 × 10^4^ sporozoites (Table 5)^20^. A three-dose regimen of 1 × 10⁴ *PbΔuis4* at 14-day intervals conferred 100% protection^20^. In *Py*, a threefold immunization of 1 × 10⁴ *PyΔuis4* also induced protection (Table 4)^23^.

**Table 5:** Overview of liver-arresting GAP breakthroughs in mice

Author	Parasite	Mice	Dose	Breakthrough
Mueller et al., 2005^20^	*PbΔuis4*	C57BL/6	5 × 10^4^ IV	33/56 (59%)
	*PbΔuis4REP*	C57BL/6	5 × 10^4^ IV	27/75 (36%)
Yu et al., 2008^29^	*PbΔFabl*	C57BL/6	1 × 10^3^ IV	5/16(31%)
	*PbΔFabl*	C57BL/6	1 × 10^4^ IV	13/15 (87%)
	*PbΔFabl*^*REC*^	C57BL/6	1 × 10^3^ IV	15/15 (100%)
	*PbΔFabl*	C57BL/6	20 mosquitoes / mice	4/5 (80%)
	*PbΔFabl*^*REC*^	C57BL/6	20 mosquitoes / mice	5/5 (100%)
Van Dijk et al., 2005^33^	*PbΔp*52	BALB/c	Not reported	1/26 (4%)
	*PbΔp*52	C57BL/6	Not reported	5/48 (10%)
Annoura et al., 2012^34^	*PbΔfabb/f-*	C57BL/6	5 × 10^4^ IV	25/25 (100%)
	*PbΔp36Δp52*	C57BL/6	5 × 10^4^ IV	3/20 (15%)
	*PbΔp36Δp52-br ‘breakthrough’*	C57BL/6	5 × 10^4^ IV	7/12 (58%)
	*PbΔfabb/f-*	BALB/c	5 × 10^4^ IV	22/25 (88%)
Annoura et al., 2014^43^	*PbΔp36Δp52*	C57BL/6	2 × 10^5^ IV	10/10 (100%)
	*PbΔb9Δp36Δp52*	C57BL/6	5 × 10^4^ IV	1/10 (10%)
	*PbΔb9*	C57BL/6	5 × 10^4^ IV	3/20 (15%)
	*PyΔb9*	BALB/c	2 × 10^5^ IV	1/8 (13%)
Vaughan et al., 2018^49^	*PyΔmei2*	BALB/cByJ	2 × 10^5^ IV	3/30 (10%)
	*PyΔmei2*	BALB/cByJ	5 × 10^5^ IV	4/30 (13%)
Franke-Fayard et al., 2022^48^	*PbΔmei2*	C57BL/6	2 × 10^5^ IV	3/10 (33%)
	*PbΔmei2-breakthrough-a*	C57BL/6	2 × 10^5^ IV	5/11 (45%)

IV: intravenous.

Due to concerns about *PbΔuis4* breakthrough infections, a double knock-out *PbΔuis3Δuis4* was created (Table 1)^24^. The parasites were completely attenuated during liver stage development. Immunizing C57BL/6 mice three times with 1 × 10^4^ *PbΔuis3Δuis4* parasites achieved sterile protection against challenge^24^, which was maintained even after rechallenge six months later (Table 4)^24^. Attempts to translate these findings to *Pf* failed because *Pfuis3* proved refractory to deletion and the syntenic gene to *uis4*, *Pfetramp10.3*, was both functionally distinct, being expressed across multiple developmental stages, and refractory to deletion^25,26^. Hence, no studies have been conducted with *uis3* or *uis4* in the context of a *Pf* GAP and attempts shifted towards other candidates.

### Single knockouts based on ΔFabB/F or ΔfabI

Further gene deletion candidates have been selected based on their essentiality during late liver stage development. Type II fatty acid synthesis (FASII) is crucial for *Plasmodium* development in hepatocytes due to the parasite’s exponential replication^27–29^. Hence, FASII-pathway members were selected for deletion, such as *fabB/F* and *fabI* (Table 1). Deletion of *fabB/F* in *Py* showed full attenuation during late liver stage development. When *PyΔfabB/F* were injected intravenously in BALB/c mice in doses up to 5 × 10^4^ sporozoites, no breakthrough infections occurred. Immunization with *PyΔfabB/F* parasites (three times 1 × 10^4^) achieved sterile protection^27^. Protection was maintained even when parasites were administered intradermally or subcutaneously, albeit at higher doses (three times 5 × 10^4^ parasites) (Table 4)^28^. Immunization studies with *PbΔfabI* mutants were never performed as injection of 1 × 10^4^ *Pb*Δ*fabI* parasites resulted in breakthrough infections in 31–100% of C57BL/6 mice (Table 5)^29^. The results of the *PyΔFabB/F* failed to translate to *Pf* as both *Pf*Δ*fabI* and *Pf*Δ*fabB/F* showed severe defects in sporozoite production and salivary gland invasion, making development of a *Pf*GAP based on these genes impossible^30^.

## Early-Arresting GAPs

### Double knock-out parasites based on Δp36Δp52 (2KO)

Other studies focused on deletion of *p52* (also referred to as *p36p*), which encodes a protein member of the 6-Cys domain family and plays an important role in the formation of the parasitophorous vacuole during liver stage development^31^. *p52* is expressed in pre-erythrocytic stages and deletion is associated with reduced parasite infectivity^31^. Immunization with *PbΔp52* induced up to 100% protection in mice (Table 4), yet in other experiments it led to breakthrough blood-stage infections in a subset of the animals (Table 5)^32,33^.

For this reason, *p36* was selected as an additional candidate for deletion (Table 1). *p36* belongs to the same 6-Cys domain family as *p52*, has an analogous function and is similarly expressed in sporozoites^31^. Simultaneous deletion of *p36* and *p52* in *Py* led to a fully attenuated phenotype, preventing breakthrough blood stage infections^31,34^. Additionally, threefold immunization with 1 × 10^4^ *PyΔp52Δp36* consistently protected against challenge in murine models (Table 4)^31^.

Given the safety and protection profile of *PyΔp36Δp52* in mice, a double knock-out *Pf*GAP, henceforth referred to as 2KO (*PfΔp36Δp52*), was generated^35–37^. *PfΔp36Δp52* failed to infect livers of mice engrafted with human hepatocytes (SCID Alb-UpA humanized mice), indicating full attenuation^36^. These results prompted approval for clinical testing.

The administration of 2KO parasites for safety testing was the first-in-human study using GAPs and occurred in six malaria-naive participants, who were exposed to escalating numbers (five and then 200) of mosquito bites (Table 2)^35^. While the first exposure to five mosquito bites was well-tolerated, one participant developed malaria on day 12 after receiving 200 mosquito bites, putting a premature end to the study (Table 6 and Table 7). Sequencing revealed that the breakthrough was caused by the incomplete attenuation of the GAP rather than by reversal to WT^35^.

**Table 6:** Overview of liver-arresting GAP breakthroughs in humans

Author	Parasite	Dose	Breakthroughs
Spring et al., 2013^35^	*PfΔp52Δp36 (2KO)*	200 mosquito bites	1/6 (17%)

**Table 7:** Overview of the protection rate of liver-arresting GAPs in humans

Author	Parasite	Dose	Challenge	Protection
Spring et al., 2013^35^	*PfΔp52Δp36 (2KO)*	5 mosquito bites	NA	NA
	*PfΔp52Δp36 (2KO)*	200 mosquito bites	NA	NA
Kublin et al., 2017^41^	*PfΔp52Δp36Δsap1 (3KO)*	150–200 mosquito bites	NA	NA
Murphy et al., 2022^42^	*PfΔp52Δp36Δsap1 (3KO)*	200 mosquito bites 3 times (2 immunizations at monthly intervals and the third 8 weeks later)	5 *PfNF54* mosquito bites 4 weeks later	3/6 (50%)
	NA	NA	5 *PfNF54* mosquito bites rechallenge 180 days later	0/3 (0%)
	*PfΔp52Δp36Δsap1 (3KO)*	200 mosquito bites 5 times (4 immunizations at 4-week intervals and the fifth 8 weeks later)	5 *PfNF54* mosquito bites 4 weeks later	4/8 (50%)
	NA	NA	5 *PfNF54* mosquito bites rechallenge 180 days later	1/4 (25%)
Roestenberg et al., 2020^45^	*PfΔb9Δslarp* (PfSPZ-GA1)	1.35 × 10^5^ cryopreserved sporozoites IV	NA	NA
	PfSPZ-GA1	4.5 × 10^5^ cryopreserved sporozoites IV	NA	NA
	PfSPZ-GA1	9 × 10^5^ cryopreserved sporozoites IV	NA	NA
	PfSPZ-GA1	4.5 × 10^5^ cryopreserved sporozoites IV three time at 2-month intervals	5 *PfNF54* mosquito bites 3 weeks later	1/12 (8%)
	PfSPZ-GA1	9 × 10^5^ cryopreserved sporozoites IV 3 times at 2-month intervals	5 *PfNF54* mosquito bites 3 weeks later	2/13 (15%)
Lamers et al., 2024^51^	*PfΔmei2* (GA2)	15 mosquito bites	NA	NA
	*PfΔmei2* (GA2)	50 mosquito bites	NA	NA
	*PfΔb9Δslarp* (GA1)	50 mosquito bites 3 times at monthly intervals	5 *Pf3D7* mosquito bites 3 weeks later	1/8 (13%)
	*PfΔmei2* (GA2)	50 mosquito bites 3 times at monthly intervals	5 *Pf3D7* mosquito bites 3 weeks later	8/9 (89%)
Roozen et al., 2025^52^	*PfΔmei2* (GA2)	50 mosquito bites	5 *Pf3D7* mosquito bites 6 weeks later	9/10 (90%)

IV: intravenous.

### Triple knock-out parasites based on Δp36Δp52Δsap1 (3KO)

To reduce the likelihood of a breakthrough, a 3KO parasite, lacking *sap1* in addition to *p36* and *p52*, was generated (Table 1)^38^. *sap1* (also referred to as *slarp*) encodes the sporozoite asparagine-rich protein 1 (SAP1) and is a post-transcriptional regulator involved in liver infectivity^39^. *PyΔsap1* parasites are completely attenuated in highly susceptible BALB/cByJ mice in doses of up to 7.5 × 10^4^ sporozoites^38^. Threefold immunization of BALB/c mice with 1 × 10^4^ sporozoites resulted in 100% protection even when challenged 210 days after the last immunization and independently of whether unattenuated parasites where administered intravenously, by mosquito bite or as blood stages (Table 4)^40^. Sequential gene deletion of *sap1* in the previously established *PfΔp36Δp52* parasite was carried out to generate *PfΔp36Δp52Δsap1*^38^. Injection with 1×10^6^ of this GAP into humanized FRG huHep mice that had been supplemented with human erythrocytes on day 7 post-infection, did not cause a breakthrough blood stage infection^38^. These observations helped obtain approval for clinical testing of the *PfΔp36Δp52Δsap1*.

The first clinical trial with the *Pf*3KO GAP saw the administration of 150–200 mosquito bites to ten malaria-naive participants to test for safety (Table 2): none of the participants developed malaria in the 28-day follow-up period^41^. A further trial assessed the protection of *Pf*3KO-immunization against challenge: malaria-naive participants were immunized by either 3 × 200 or 5 × 200 *Pf*3KO mosquito bites, followed by CHMI four weeks later^42^. The protection rate was 50% in both groups after CHMI, demonstrating that additional immunizations beyond three times does not result in higher protection rates (Table 7). To investigate duration of protection, a second CHMI of protected participants was carried out six months after the last immunization. Protection was 15% in both groups after the second CHMI, indicating that immunity waned over time (Table 7)^42^.

### Double knock-out based on Δb9Δslarp (GA1)

Another early-arresting GAP was based on genetic deletions of *b9* and *slarp* (also referred to as *sap1*). *b9* also encodes a member of the *Plasmodium* 6-Cys family (Table 1)^39,43^. Breakthrough blood stage infection was absent upon injection of Swiss or BALB/c mice with 5 × 10^4^ single-deletion mutant *PbΔb9*. However, 20% of C57BL/6 mice became parasite-positive after inoculation with 5 × 10^4^ *PbΔb9* sporozoites (Table 5)^43^.

Based on these results, a double knock-out was generated, i.e. *PbΔb9Δslarp*. No breakthrough infection occurred after injection of 2 × 10^5^ *PbΔb9Δslarp* sporozoites. Immunization of BALB/c mice with as few as 1 × 10^3^ sporozoites induced protection against challenge with WT sporozoites. In C57BL/6 mice three immunizations with 1 × 10^4^ sporozoites were required to induce protection, which was maintained for at least 180 days (Table 4)^44^.

Subsequently, a *Pf*GAP, *PfΔb9Δslarp*, henceforth referred to as GA1, was generated. Survival within hepatocytes in vitro was completely abrogated by day 4 and parasites were absent at day 5 when assessed in immunodeficient SCID Alb-uPA mice engrafted with human hepatocytes^44^.

The promising results of the preclinical experiments warranted further assessment of GA1 in humans (Table 2). The biotechnology company Sanaria produced the GAP as an injectable cryopreserved product, referred to as PfSPZ-GA1. The PfSPZ-GA1 product was deemed safe, as no blood stage infections were observed after administering up to 9 × 10^5^ sporozoites to humans^45^. Immunizations of malaria-naive participants were carried out three times at two-month intervals with either 4.5 × 10^5^ or 9 × 10^5^ sporozoites and followed by homologous challenge with five *Pf*NF54-infected mosquito bites three weeks later^46^. Three of the 25 PfSPZ-GA1 immunized participants were protected against CHMI, one in the low-dose (4.5 × 10^5^ sporozoites) and two in the high-dose (9 × 10^5^ sporozoites) group (Table 7)^45^.

## Late Arresting GAPs

It had been previously demonstrated that prolonging GAP survival in the liver could improve protection rates through increased antigenic exposure^28^. The knowledge that CPS, where the concomitant administration of live unattenuated sporozoites with antimalarial agents causes the parasites to die just after reaching the blood stage, achieved the highest protection (100%)^16,17^ of all WSp candidates to date corroborated this hypothesis by confirming the importance of prolonged antigenic exposure for protection in humans.

### Single knock-out GAP based on Δmei2

The gene meiosis inhibited 2 (*mei2*) was identified as a promising target for a late-arresting GAP (Table 1)^47,48^. *mei2* encodes an RNA-binding protein expressed only during liver stage development and is involved in DNA segregation^47^. Removal of *mei2* resulted in a late-arresting phenotype with parasites incapable of producing infectious merozoites^47^. A breakthrough rate of 10% and 13% was observed in BALB/cByJ mice after intravenous administration of 2 × 10^5^ and 5 × 10^5^ sporozoites, respectively^49^. Similar results were reported for *Pb,* with C57BL/6 mice becoming blood stage patent after administration of 2 × 10^5^ sporozoites (Table 5)^48^. Immunizing BALB/c mice three times with 1 × 10^4^ *PyΔmei2* sporozoites resulted in 100% protection against challenge, showing promise as a late-arresting GAP (Table 4)^47^.

The *PfΔmei2* was created by two different research groups and termed GA2 or late arresting replication competent 1 (LARC1) (Table 2)^48,50^. *PfΔmei2* parasites exhibited normal infection of human hepatocytes in vitro for up to nine days^48,50^. When FRG huHep mice were infected with 1 × 10^6^ GA2 or LARC1 sporozoites and supplemented with human erythrocytes, parasites were undetectable in blood from GAP-infected mice for up to 28 days post-infection^48,50^. The lack of parasitic genetic material in blood of GA2-infected mice and the absence of their growth in vitro suggests that either parasites are cleared in the liver stage therefore never entering the bloodstream or that aberrant, non-viable merozoites are released from the liver yet are incapable of infecting erythrocytes^48,50^. Based on these findings, GA2 was approved for clinical testing (Table 2).

In the first part of a phase 1/2a trial, which aimed to confirm the attenuation phenotype and safety profile of GA2, two sequential cohorts were exposed to 15 and then 50 GA2-infected mosquito bites (Table 2)^51^. No blood stage infections occurred, and adverse events were limited. The second part saw the three-fold immunization of ten participants with 50 GA2-infected mosquito bites at 28-day intervals with a homologous CHMI three weeks after the last immunization. Controls consisted of GA1- and placebo-immunized participants. Efficacy of GA2 was 89%, whereas only 13% of GA1- and none of the mock-immunized participants were protected (Table 7)^51^, demonstrating for the first time in humans that late-arresting GAPs are superior to early-arresting GAPs.

Next, GA2-immunized participants (*n* = 10) and placebo-exposed participants (*n* = 5) were exposed once to 45–55 mosquito bites and challenged in a CHMI with five 3D7-infected mosquitoes six weeks later. 90% of the GA2-exposed individuals were protected and did not develop blood stage parasitemia (Table 7), whilst all placebo-exposed participants became parasite-positive. This indicates that a single dose of GA2-parasites with 50 mosquito bites is sufficient to elicit 90% protection in a small cohort of individuals^52^.

### Double knock-out based on Δmei2Δlinup (LARC2)

Since *PyΔmei2* parasites were incompletely attenuated when administered in high doses to susceptible BALB/cByJ mice (Table 5)^49^, a double gene deletion mutant was generated in the form of *PyΔmei2Δlinup* (LARC2) (Table 1). LARC2 may have similar immunogenicity as GA2, but an enhanced safety profile. *linup* encodes the liver stage nuclear protein, which is a conserved protein localizing to the nucleus in liver stage *Plasmodium* parasites^53^. Single deletion mutant *PyΔlinup* showed that the gene is essential during late liver stage development^53^. Injection of susceptible BALB/cByJ with 2 × 10^5^ or 5 × 10^5^ double deletion mutants *Py*LARC2 parasites did not cause breakthrough infections^54^. *Py*LARC2 elicited durable protection with 90% of BALB/cJ mice being protected when immunized twice with 5 × 10^4^ *Py*LARC2 sporozoites at one-month intervals and subsequently challenged six months later^54^. Administration of 5 × 10^4^ cryopreserved sporozoites at two-week intervals was equally protective. Finally, animals immunized with *Py*LARC2 completely cleared the infection within 12 days upon injection of blood stage parasites (Table 4)^54^.

In the same study, *Pf*LARC2 was described (Table 2)^54^. To assess the risk of breakthrough infections in vivo, FRG NOD huHep mice were injected with one million cryopreserved *Pf*LARC2 or WT parasites and then supplemented with human erythrocytes six and seven days after infection. Blood collected from *Pf*LARC2-injected mice was parasite-negative up to experiment termination, in contrast to blood cultures from WT-injected mice that became parasite-positive on days 1–3 after transition^54^. These promising results prompted the approval for clinical testing of aseptic cryopreserved PfSPZ-LARC2. Current planning of clinical trials is underway to start testing *Pf*LARC2 in healthy participants as well as in individuals in endemic countries (Table 3).

## Possible immunological mechanisms of protection

Both humoral and cellular immune responses play a role in protection against malaria, with CD4^+^ and CD8^+^ T-cells being important for establishing liver immunity^55^. Late-arresting parasites may be more efficient at activating and recruiting liver-resident CD8^+^ T-cells through increased antigenic exposure. Rodent work has shown that immunization with late-arresting *PyΔFabB/F* induced a higher absolute count of CD8^+^ T cells that also persisted for a longer period of time compared to early arresting RAS^28^. Analysis of immune responses from C57BL/6 mice that had been immunized twice with 5 × 10^4^ late-arresting *Py*LARC2 sporozoites showed that CD8^+^ T-cells and specifically CD69^hi^CXCR6^hi^ CD8^+^ T_RM_ cells were significantly increased in immunized mice compared to controls^54^.

A correlation between CD8^+^ T-cells and protection against malaria has been reported in human studies as well^17,56–58^. Interferon-γ (IFN-γ), tumor necrosis factor-α (TNF-α) and interleukin-2 (IL-2) producing CD4^+^ T cells and, to a lesser extent, CD8^+^ T were increased after immunization with early-arresting 2KO parasites. These responses exhibited a dose-dependent pattern, with significant increases after 200 mosquito bites compared to 5 mosquito bites^35^. Similar findings were reported after immunization with GA1: the frequency of IFN-γ producing CD4^+^ and CD8^+^ T cells was significantly increased after three immunizations, yet responses did not correlate with protection^45^.

Immunization with GA2, whether it happened once or multiple times, resulted in an increased frequency of CD4^+^ and Vδ2 γδ T-cells with a memory phenotype, preferentially expressing IFN-γ, TNF-α and IL-2^51,52,59^. However, circulating CD8^+^ and NK cells were not increased upon immunization^44,45^. The difficulties in detecting CD8^+^ T cells may not necessarily disprove their involvement in antimalarial immunity. As *Pf*-specific CD8^+^ T-cells may exhibit liver-resident phenotypes, they are undetectable in peripheral blood and therefore difficult to identify^60^. Liver-residency of immune cells would also explain the superior protection of late-arresting compared to early arresting GAPs^28,51^, as their prolonged permanence in the liver allows for the recruitment of tissue-resident CD8^+^ T cells^28^.

Humoral immune responses also contribute to protection against malaria^55^. Anti-CSP antibodies are frequently measured as they are directed against the most abundant protein on the sporozoite surface^61^. Indeed, all reviewed studies found an increase in CSP titres in humans after immunization^41,42,45,51,52,59^, which however did not correlate with protection^42,45,51,52^. Interestingly, serum from 3KO-immunized participants was able to block in vitro hepatocyte traversal and invasion^35,36^, suggesting inhibitory capacity of formed antibodies in immunized participants. This was confirmed in vivo by administering serum from participants to FRG huHep mice^35^. Subsequent challenge with WT parasites showed an 88% reduction of liver stage burden. The serum samples with the highest functional capacity in vivo had the lowest anti-CSP antibody titers, suggesting other sporozoite proteins may be important in hepatocyte invasion and subsequent development^41^.

Timing of GAP arrest may also play a role in antibody production: when humoral responses were compared between GA1- and GA2-immunized participants, more and different antigens were detected in the GA2-immunized group, indicating increased antigenic exposure during late liver stage development^59^. The exact role of antibodies in liver immunity remains to be elucidated, although they interfere with parasite movement, traversal and hepatocyte invasion^55^.

## Discussion

This review presents the currently existing *Pf*GAPs, including research that was instrumental to their generation. These novel malaria vaccine candidates should be further explored, and specifically questions regarding biosafety, large scale production and implementation need to be addressed.

### Biosafety

Safety is key for the implementation of any GAP. Despite extensive preclinical testing, instances of incomplete attenuation have led to hesitancy for the large-scale use of GAPs^35^. Breakthrough infections in rodent malaria species may not always predict a similar phenotype in humans, as blood stage infections were detected in highly susceptible BALB/cByJ mice after administration of 2.5-5 × 10^5^ *PyΔmei2* sporozoites^49^, but not for *PfΔmei2* in humanized mice and preliminary clinical trials^48,50^. Further testing in larger groups may be necessary to define the risk of breakthroughs in clinical settings. However, if the chance of such an event occurring is infinitesimal, it may not be detected in phase 2 and 3 clinical trials and still can never be proven to be absent. Hence, humanized mice injected with human erythrocytes remain the best approximation for safety predictions before clinical testing in humans.

Biosafety concerns are pertinent to the use of genetically modified organisms (GMOs) in medicine, with varying regulations worldwide. One concern is escape from the controlled conditions of the laboratory, potentially disrupting ecosystems. All GAPs created so far arrest prior to reaching the blood stage, making propagation past the liver and transmission to other humans and mosquitoes effectively impossible^41,42,44,48,50^. Furthermore, no transposable genetic elements have been identified in *Plasmodium*, meaning that DNA cannot spontaneously be exchanged between two parasites unless it happens during sexual reproduction, a process only occurring in the mosquito midgut^62^.

Another voiced concern is that of horizontal gene transfer from GMOs to host cells^63^. However, in the case of GAPs, vectors are not self-mobilizable and not present in the vaccine product. Removal of all exogenous genetic material used to guide the deletion in the GAP means that no new DNA is being introduced in the host^18,44,48^. GMO use in medicine is not novel, adenovirus-based vaccines for SARS-CoV-2 and Ebola are genetically engineered^64^. Given the proven success of such vaccines, GAPs share similar potential.

### Protective capability

**Fig. 1:**
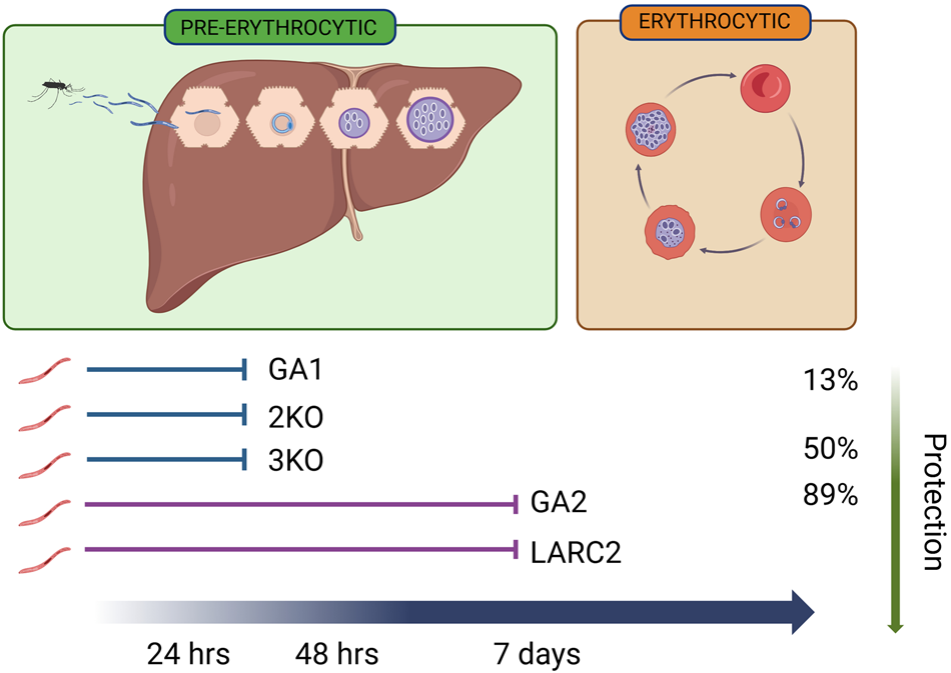
Schematic representation of liver stage arresting GAPs that have or will soon be tested in clinical trials. Length of development of each GAP in relation to protection in humans is shown. Candidates include the early arresters GA1 (*PfΔb9Δslarp*), 2KO (*PfΔp36Δp52*) and 3KO (*PfΔp36Δp52Δsap1*). GA2 (*PfΔmei2*) and LARC2 (*Pf Δmei2Δlinup*) are late arresters.

For any GAP to implemented, it must be safe but also confer high protection in malaria-endemic areas, where it may be hampered by vaccine hyporesponsiveness^65^. Most GAPs have achieved lower than desired efficacy and have therefore not proceeded to further clinical testing^35,45,66^. However, the advent of late-arresting GAPs (i.e. GA2 and LARC2), open the road to new scientific possibilities. The direct comparison of GA2 with GA1 unequivocally confirmed the importance of the late liver stage in achieving protection against malaria^51^ (Fig. 1). An important question is whether late-arresters maintain protection rates against heterologous challenge. If protection were lower than desired, one possibility would be to generate GAPs with different genetic backgrounds to match the parasites in circulation. This approach is limited, as the number of existing *Pf* strains is considerable and new ones continuously evolve^67^. Improving protection over time could require boosts, although the frequency and timing remains to be determined. Duration of protection also needs to be assessed. In this regard results from CPS studies conducted in malaria-naive individuals show potential with protection lasting up to two years after immunization^68^. Despite the many open interrogatives, the superior protection of GA2 compared to previously developed early-arresting GAPs is promising and makes late liver stage arresting parasites potential candidates for further vaccine development and large-scale implementation.

### Implementation of GAPs

Another important question pertains to the feasibility of introducing GAPs as a large-scale vaccination strategy. The first potential obstacle is the cost of manufacturing of cryopreserved sporozoites, as it still requires rearing of aseptic mosquitoes, manual dissection of salivary glands and extraction of sporozoites. However, dissection-independent production of sporozoites^69^ and in vitro culturing of sporozoites has been reported^70^, allowing a complete bypass of the dissection step. Storage of sporozoites at −150 °C could pose logistical challenges in endemic areas, but liquid nitrogen systems already exist in Africa and require minimal infrastructure. The additional advantage of a liquid nitrogen-based cold chain is that, except for the generation of liquid nitrogen, it is independent of electricity supply^71^. While logistical hurdles exist, recent technological advances offer viable solutions.

### Future Directions: Remaining Problems and Potential Solutions

Despite the promise of efficacy and deliverability of GAPs, some questions remain open. The route of administration of GAPs is one such discussion point. Intravenous injection of sporozoites is more efficacious as large proportions of sporozoites reach the liver^72^, yet intradermal and/or intramuscular administration would be more practical^73^. Intradermal or intramuscular injections could also provide the advantage of early immune system activation^74,75^, as skin-draining lymph nodes play an important role in the initiation of the immune response against malaria^76^.

Another question is whether approved malaria vaccines may act synergistically or antagonistically with GAP vaccine candidates. If part of the African population is immunized with RTS,S and R21, could efficacy of GAPs be decreased due to pre-existing immunity? The two subunit-based malaria vaccines induce mostly humoral immunity targeted to CSP. However, since antibodies do not distinguish between GAPs or WT parasites, GAPs may be intercepted before reaching the liver where important immune responses occur. Further clinical studies involving GAPs in combination with RTS,S or R21 are necessary to understand the interactions between the existing vaccines.

## Conclusion

In conclusion, GAPs have shown to be safe, well-tolerated and highly efficacious. What emerges from this review is that any future research should focus on late-arresting GAPs, as they have achieved the highest protection rates in malaria-naive individuals (Table 3, Fig. 1). While the results of phase 1/2 clinical trials are promising, some aspects remain to be investigated: efficacy of all late-arresting GAPs must be confirmed in larger trials, against heterologous challenge and in pre-exposed populations; duration of protection needs to be examined also. Despite these questions being unanswered to date, this field of research is just beginning to evolve. Many of the current hurdles may be easily overcome soon, making GAPs increasingly promising vaccine candidates against malaria.

## Data Availability

No datasets were generated or analysed during the current study.
